# Proteomic study of aqueous humour in diabetic patients with cataracts by TMT combined with HPLC-MS/MS

**DOI:** 10.1186/s12886-023-03162-2

**Published:** 2023-10-26

**Authors:** Weihai Xu, Ya Liang, Yunxia Zhu, Tong Sun, Zhilan Yuan, Xiao Han

**Affiliations:** 1 Department of Ophthalmology, Binhai county people’s hospital, Yancheng, Jiangsu China; 2 Department of Ophthalmology, the First Affiliated Hospital, Nanjing Medical University, Nanjing, Jiangsu China; 3 Key Laboratory of Human Functional Genomics of Jiangsu Province, Jiangsu Diabetes Center, Nanjing Medical University, Nanjing, Jiangsu China

**Keywords:** Cataract, Diabetes, Aqueous humour, TMT, Bioinformatics

## Abstract

**Background:**

The purpose of this study is to identify the proteomic differences between the aqueous humour of diabetes patients with cataracts and that of non-diabetic sufferers of cataracts in a clinical setting.

**Methods:**

Patients were divided into the diabetic experimental group and the non-diabetic control group. Aqueous humour specimens were obtained via cataract surgery. Sample proteins were treated with a TMT reagent, separated using a cation chromatography column, and analysed using a C18 desalting column. Proteins were identified using HPLC-MS/MS. The differential proteins were identified using both a p value of < 0.05 and a fold change of > 1.2. GO classification enrichment analysis, KEGG pathway enrichment analysis, protein interaction network analysis, and ingenuity pathway analysis were all carried out. The expression level of four differential proteins were verified by Western blot, and GC and TTR expressions were further examined using an expanded sample pool.

**Results:**

The postprandial glucose levels between the experimental group (9.40 ± 1.35 mmol/L) and the control group (6.56 ± 0.81 mmol/L) were significantly different, with a p value of 1.16E-06. It is important to note, however, that the baseline levels of the parameters showed no statistical differences. In total, 397 aqueous humour proteins were identified; of these, 137 showed significant differences, with 63 upregulated ones and 74 down-regulated ones. The differential proteins play important roles in numerous biological processes and pathways, such as complement and coagulation cascades (p = 1.71E-09). Some of these differential proteins are associated with diabetic retinal degeneration and other diabetic complications. Differential proteins, such as HP, GC, and TTR, have high node degree in the protein interaction network. Western blot results further confirmed that GC were down-regulated while TTR was up-regulated in aqueous humour under diabetic condition.

**Conclusion:**

A list of differential proteins in the human aqueous humour of diabetic patients was established. Proteins with high interaction scores as per protein interaction analysis, such as GC and TTR, were further verified and could potentially be used as early diagnostic markers for diabetic eye complications in clinical practice.

**Supplementary Information:**

The online version contains supplementary material available at 10.1186/s12886-023-03162-2.

## Introduction

Diabetes mellitus is a condition in which either the pancreas no longer produces enough insulin or cells stop responding to the insulin that is produced such that glucose in the blood cannot be absorbed into the cells of the body. Symptoms include frequent urination, lethargy, excessive thirst, and hunger while treatment options include changes in diet, oral medications, and, in some cases, daily injections of insulin. The prevalence of diabetes has been shown to be correlated with both lifestyle choices and aging. With the influence of lifestyle and aging, the incidence of diabetes increased rapidly. The number of diabetics in the world has increased from 151 million in 2000 to 537 million in 2021 and is expected to reach 783 million by 2045, making the global prevalence of diabetes 11.2% [1]. Diabetes could cause cataracts [2, 3]; several clinical studies have shown that diabetic patients often suffer from ocular complications, especially severe vascular complications such as diabetic retinopathy [4–7]. However, the ways in which diabetes affects the ocular tissues and organs in the early stages of the disease, which lead to cataracts, remain unclear.

An increasing number of studies have found that changes in the protein composition and content of the aqueous humour are related to the occurrence and development of numerous ocular diseases, including diabetic retinopathy, age-related macular degeneration, and myopia, among others. the ways in which changes in aqueous humor proteins are related to the formation of cataracts in diabetic patients remain unclear [8, 9].

Proteomics is the study of a proteome, that is, the ensemble of proteins produced by either an organism, a system, or its subpart, and of the interactions between proteins within the system. Today, proteomics studies have been conducted on many eye diseases, including diabetic cataracts, diabetic retinopathy, and glaucoma. These studies uncovered the pathogeneses and new diagnostic markers of the diseases, which paved the way for the development of new diagnostic and therapeutic options [10–12].

High performance liquid chromatography (HPLC) uses a high-pressure infusion pump to insert a specified mobile phase into a chromatographic column filled with an agent that separates and determines the test substance. TMT (tandem mass tag) is a relative, absolute quantitative technology developed by Thermo Fisher Scientific for isotope labelling of polypeptides. It can simultaneously compare the protein expression of up to 16 samples through serial analysis by high-resolution mass spectrometry, such as high-performance liquid chromatography, by specifically labelling the amino groups of polypeptides. In this study, TMT, in combination with HPLC-MS/MS, was used for the proteomics study of aqueous humour samples obtained from either diabetes patients with cataracts patients or non-diabetic cataract patients. Bioinformatic analysis was used to identify the differential protein composition. This study provides a theoretical basis for the examination of the pathogenesis of diabetes-induced cataracts, leading to the potential discovery of early diagnostic markers of diabetic eye complications.

## Materials and methods

### Inclusion of patients

The patients were divided into an experimental group (patients with both diabetes and cataracts) and a control group (patients with age-related cataracts). The inclusion criteria for the patients were as follows: (1) Patients diagnosed with diabetes and cataracts with fasting blood glucose levels of < 8.0 mmol/L controlled by oral metformin administration before surgery. (2) No significant abnormalities on the anterior segmental examination, except for lens opacity. (3) Trauma, glaucoma, uveitis, and fundus lesions were excluded. (4) No history of other systemic drug intake. All enrolled clinical samples were from surgical patients who underwent phacoemulsification extraction. Informed consent was obtained from both patients and their families. This study was approved by the hospital ethics committee and was conducted in accordance with the Declaration of Helsinki.

### Sample collection and processing

A total of 20 diabetic patients (20 eyes) with cataracts and 21 patients with age-related cataracts (21 eyes) were enrolled in this study.

The patients were prepared prior to cataract surgery; the ocular surface and the conjunctival sac were rinsed with sterile saline, and the fluid in the conjunctival sac was aspirated. Using a 1 ml pinpoint-removed disposable syringe (30G needle), the anterior chamber was penetrated from 0.5 mm in front of the 2 o’clock corneal limb parallel to the iris plane. After 0.1–0.2 ml of aqueous flow was pumped out of the chamber, the needle was removed. The puncture step was conducted carefully to avoid injuring either the iris, lens, or corneal endothelium.

After the aqueous humour was removed, the cataract surgery proceeded. Aqueous humour samples were placed in sterile collection tubes, centrifuged at low speed, and stored in -80 °C until further analysis could be conducted.

Three specimens from each of the two groups were randomly selected and combined to achieve the requirement of more than 300 µg of protein with a protein concentration of not less than 1 µg/µl. A total of six combined sample groups were obtained; groups 1–3 were the control group and groups 4–6 were the experimental group. A portion of each sample was used for Western blot analysis.

### Protein extraction and quantification

The ProteoMiner kit (Bio-Rad, Cat. #1633006) was used to both remove the high-abundance proteins from the aqueous humour samples and concentrate the low and medium-abundance proteins. The enriched aqueous humour protein samples were treated with 10 mM DTT for 1 h under 56 °C. The samples were then treated with 55 mM of IAM for 1 h in the dark.

Pre-cooled acetone was added at four times the volume of the sample solution. The samples were then precipitated at -20 °C for over 3 h and centrifuged for 20 min at 20,000 g at 4 °C; precipitate was then collected. Three hundred microliters of reconstitution buffer were added, and the samples were then sonicated for 3 min. Proteins were quantified using the BCA protein assay kit (Nanjing Vazyme Biotech Co. Ltd, Cat. # E112).

### Sample trypsinization

One hundred microgram of protein was taken from each sample and treated with TEAB solution (50% TEAB, containing 0.1% SDS). 3.3 µg of trypsin (1 µg/µl) was then added to each sample, which was then incubated at 37 °C for 24 h. Afterwards, another 1 µg of trypsin was added before another incubation period at 37 °C for 12 h. Samples were lyophilized and 30 µl of TEAB solution was used to reconstitute the peptide fragments. The digestion efficiency was assessed using the MALDI Tof/Tof method.

### Labelling of protein peptides and mixing of samples

The labelling reagent was equilibrated at room temperature, and isopropyl alcohol was added to each sample containing the labelling reagent. The samples were then vortexed, centrifuged, and left at room temperature for 2 h before being vacuum dried. The contents of the TMT-reagent-labelled tubes of the same group were combined.

### Strong cation exchange chromatography

The column was washed with MilliQ water for 10 min, and the pressure was balanced. Pump A was filled with solution A (25% can and 10 mM KH_2_PO_4_; the pH was adjusted to 3.0 with phosphoric acid), and pump B was filled with solution B (25% ACN, 2 M KCL, and 10 mM KH_2_PO_4_; the pH was adjusted to 3.0 with phosphoric acid). Solution A was balanced for 10–20 min at a flow rate of 1 ml/min. The samples were then injected into the loop, and the system was equilibrated with solution A for 10 min. Then, both pump A and pump B were flushed with MilliQ water until both the absorption peak and the pressure levelled off.

### Purification of peptide fragments

The column material was activated using 1 ml of methanol with a flow rate of 2–3 drops/second; the sample was equilibrated with 5% ACN at a flow rate of 1 drop/second. The samples were dissolved with 1 ml of MilliQ water over the column, and the column was washed with 1 ml of 5% acetonitrile. The column was twice eluted by 500 µl of pure acetonitrile at a flow rate of 1 drop/second. Acetonitrile was drained using low temperature centrifugation, and the peptide was redissolved with 0.1% formic acid.

### HPLC-MS/MS analysis

The peptide signals were detected using a Thermo-Fisher Q-Exactive high-precision liquid mass spectrometer at a flow rate of 300 nl/min through the analytical column. The resolution of the primary mass spectrometer was set to 70,000 at 200 m/z and a scanning range of 300–1800 m/z while the resolution of the secondary mass spectrometer was set to 35,000 at a scanning range of 200 m/z; the normalized collision energy was 30 eV, and the acquisition standard was 10 peak intensities.

### Protein identification and quantitative analysis

Mascot 2.3 and Proteome Discoverer 1.4 were used to identify and quantify the raw mass spectrometry data. The accuracy of the primary mass spectrometry used for qualitative analysis was 15 ppm while the accuracy of the secondary mass spectrometry was 20 ppm; the maximum allowable number of missed cleavages during enzymolysis was one amino acid. The Swissprot human library was used as the protein database. The peptide values used for quantification followed the midposition method; the minimum number of unique peptides used for quantification was one. The median value of all quantifiable proteins was selected in a set of samples for correction. The *p*-value was set to less than 0.05 and the fold change in the direct ratio of the intensity of the reporter group was set to more than 1.2. Proteome data were processed using Proteome Discoverer 1.4 and MS/MS spectra were retrieved from the UniProtKB / Swiss-Prot human database. The false discovery rate (FDR) was set to < 0.05. The relative quantification of peptides was determined on the basis of the relative intensities of the reporter ions released from TMT-labelled peptides.

### Clustering heat map analysis

Using an R package (Version 3.4), a hierarchical clustering heat map was generated to show the expression trends of differential proteins between the two groups. Quantitative information was normalized to the interval (-1,1).

### GO and KEGG annotation and enrichment analysis

GO functional enrichment analysis was performed on the target protein collection using Blast2GO (https://www.blast2go.com/). The process includes sequence alignment (BLAST), GO entry extraction (Mapping), GO annotation (Annotation), and InterProScan supplementary annotation (Annotation Augmentation). KAAS (KEGG automatic annotation server, https://www.genome.jp/tools/kaas/) was used to perform the annotation analysis of the KEGG signalling pathway in the target protein collection. The KEGG database (https://www.genome.jp/kegg/) was used for enrichment analysis of the pathway [13–15]. Fisher’s exact double-end test was used to test for differentially expressed proteins in the context of the identified proteins. A *p* value of < 0.05 was considered statistically significant. These pathways were classified according to the KEGG website pathway hierarchy classification method.

### Protein-protein interaction network analysis

The connections between proteins were analysed according to STRING (http://string-db.org/). The generation and analysis of protein interaction networks was performed using CytoScape 3.9.1.

### Ingenuity pathway analysis

A list containing all differentially expressed proteins, and the fold change values, ​​was uploaded to the IPA software (QIAGEN) for analysis. The IPA software is based on the Right-Tailed Fishers Exact Test algorithm, which can analyse the association of the related proteins with the disease models.

### Western blot assay

The Western blot assay was performed following a standard process. Briefly, a total of 30 µg of each sample was taken for SDS-PAGE electrophoresis. The membrane was blocked with 5% BSA and incubated with a primary antibody at a manufacturer-suggested concentration overnight at 4 ℃ on a shaker. The next day, the membrane was washed with TBST and incubated with a secondary antibody (1:4000) for 1 h at room temperature. The bands were defined using a chemiluminescence imaging analyser. Band intensity was quantified using Image J (NIH) software. Coomassie Blue was used as an internal control.

### Research reagents

The TMT labelling kits were purchased from Thermo (Cat. #90061) and BCA kits were purchased from Nanjing Vazyme Biotech Co. Ltd (Cat. #E112). Rabbit CRYGS antibody was purchased from Abclonal (Cat. #A7888). Haptoglobin antibody (HP) (Cat. #16665-1-AP), transthyretin antibody (TTR) (Cat. # 11891-1-AP), and the vitamin D binding protein antibody (GC) (Cat. # 16922-1-AP) were purchased from Proteintech.

### Statistical analysis

A two-sample, two-tailed *t*-test was used to compare protein expressions; the difference was considered statistically significant when the *p* value was less than 0.05 and the fold change was greater than 1.2; other data were analysed using GraphPad Prism 9.0. The non-parametric *t*-test was used to analyse and compare the statistical significance of the two groups. A *p*-value of less than 0.05 indicated a statistically significant differences, all data were expressed as mean ± SEM.

## Results

### Clinical information on patients

There were no statistically significant differences between the two groups in terms of general clinical information including gender, age, visual acuity, degree of lens opacity clouding, and intraocular pressure. There was a significant difference, however, in postprandial blood glucose between the two groups with a *p*-value of 1.16E-6. No obvious fundus neovascularization or insulin therapy was observed during treatment. With the exception of blood glucose, the baseline levels of the two groups were consistent and could be used in this comparative study. The patients’ characteristics are summarized in Table 1.

**Table 1:** Clinical Characteristics of the Study Patients

Item	Study group	Control group	*p* value
Operated eyes	20	21	
Gender			0.658
Male	8	7	
Female	12	14	
Average age (years)	65.1 ± 11.2	68.7 ± 9.4	0.432
Vision	0.2 ± 0.18	0.25 ± 0.12	0.213
Intraocular pressure (mmHg)	14.9 ± 1.7	14.3 ± 4.9	0.456
Fasting blood glucose (mM/L)	5.66 ± 1.15	5.34 ± 0.77	0.42
Postprandial blood glucose (mM/L)	9.40 ± 1.35	6.56 ± 0.81	1.16E-06

### Identification of differential proteins in aqueous humour

We first quantified our sample concentration. The protein concentration of the samples ranged from 2.1 to 6.3 µg/µl, with total protein content ranging from 426 to 1512 µg (Table 2). Mascot software was used to analyse the charge number and mass-to-charge ratio of the parent ions for each spectrum. A total of 4152 spectrum models were paired by database comparison; of these, 1182 were specific ones. A total of 2121 peptides were paired; of these, 746 peptides were unique. In total, 387 proteins were identified. After applying the criteria of *p* < 0.05 and fold change > 1.2, 137 proteins were defined as differential proteins; of these, 63 were up-regulated and 74 were down-regulated.

**Table 2:** Protein Concentration and Contents of the Samples

Sample No.	Concentration (µg/µl)	Approximate volume(µl)	Total protein (µg)	
1	2.1	200	426	
2	2.3	200	450	
3	3.2	200	648	
4	3.0	280	848	
5	3.3	240	792	
6	6.3	220	1386	

Aqueous humor extracts from the sample group were combined into 3 experimental samples (Sample 4–6) and 3 control samples (Sample 1–3)

### Bioinformatic analysis and proteomic annotation of differential proteins

**Fig. 1:**
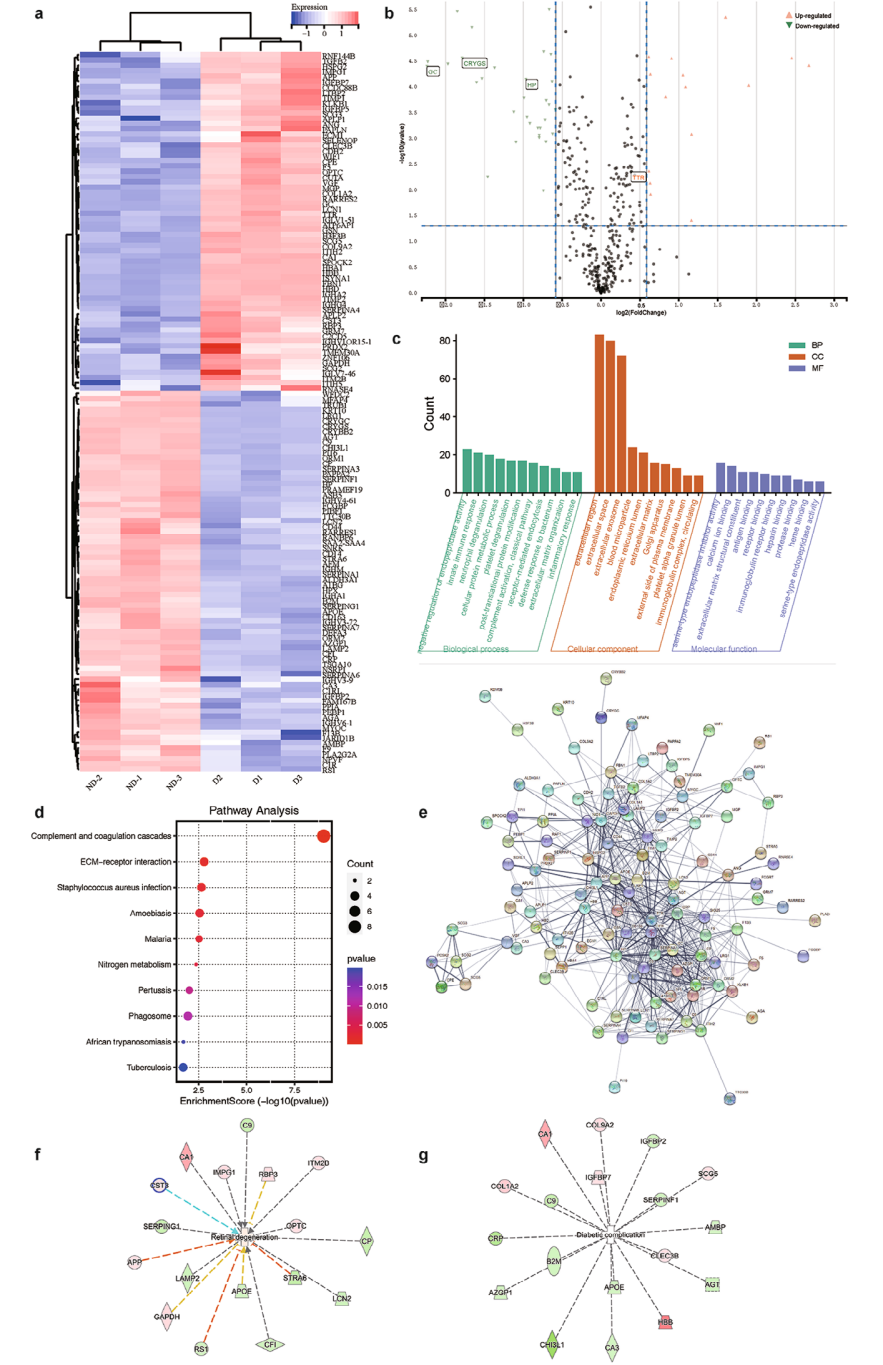
**Proteomic analysis of the differential proteins**. (**a**) Hierarchical clustering analysis. D1, D2, and D3 are the diabetes-related cataracts group; ND1, ND2, and ND3 are the common cataracts group. Red represents high abundance while blue represents low abundance. The quantitative information was normalized to the (-1,1) interval. (**b**) Volcano plot analysis. The horizontal blue dotted line represents -log_10_0.05 while the vertical blue dotted lines represent ± log_2_1.2. The green downward triangles indicate the downregulated differential proteins while the red upward triangles indicate the upregulated differential proteins. (**c**) GO enrichment analysis. The Y-axis represents the number of differential proteins contained in the corresponding category. (**d**) GO pathway enrichment analysis. The dots in the figure represent the indicated signalling pathways. The size of the dots represents the number of differential proteins contained in the pathway. The colour of the dots represents the *p* value of the pathways, with red indicating a low *p* value and blue indicating a high *p* value. The horizontal position of the dots represents the pathway enrichment scores. (**e**) Protein interaction network analysis. The MCC (maximal clique centrality) algorithm was used to derive the score for each pivotal protein through the cytohubba plug-in of Cytoscape. The larger the score, the greater the weight of the node protein on the whole network. (**f**) Differential proteins associated with retinal degeneration. (**g**) Differential proteins associated with diabetic complications

The hierarchical cluster plot and the Volcano plot for the differential proteins were then constructed (Fig. 1a, b). For the cluster plot, we first clustered D-1 and D-3 into one class and re-aggregated with D-2 into class D (diabetes patients with cataracts). Similarly, ND-1 and ND-3 were first clustered together and then clustered with ND-2 to form the ND class (common cataract group). It showed that the abundance of protein in the same class were relative concordant. However, the expression pattern of the listed proteins in class D and class ND showed obvious differences.

GO functional enrichment analysis sorted the differentially expressed proteins according to biological process, cellular components, and molecular function. For the categories of biological process and molecular function, the top-ranking terms into which differential proteins fell were negative regulation of endopeptidase activity and serine-type endopeptidase inhibitor activity, respectively (Fig. 1c). For the category of cellular components, most of the differential proteins were shown to be either extracellular proteins, extracellular space proteins, or extracellular exosome proteins.

The results of the analysis of the KEGG pathway showed that the main pathways enriched by differential proteins fell into the categories of complement and coagulation cascades, ECM receptor interactions, and staphylococcus aureus infection, among others; of these, the complement and coagulation cascade pathways showed the most significant differences with *p* = 1.71E-09 (Fig. 1d).

The protein-protein interaction network of the differential proteins was explored (Fig. 1e). The 20 nodes with the highest number of interactions were HP (31), GC (30), SERPINA1 (30), TTR (30), HPX (28), CP (26), CRP (26), AMBP (24), APOE (24), APP (24), ORM1 (24), AFM (23), GAPDH (23), GIG25 (22), ITIH2 (22), TIMP1 (22), CST3 (21), ORM2 (20), B2M (18), and C9 (18).

To explore the relationships between differential proteins and human eye-related diseases, we performed ingenuity pathway analysis. The results showed that proteins like CST3, C9, and ITM2B, among others, are associated with retinal degeneration (Fig. 1f) while proteins such as COL1A2, IGFBP2, and AGT, among others, are associated with diabetic complications (Fig. 1g).

### Protein expression validation

The results of both the quantitative mass spectrometry and interaction network analysis led to the selection of the three functional proteins with the most significant integral values, namely HP (Haptoglobin), GC (vitamin D-binding protein), and TTR (transthyretin). We also included the eye-specific differential protein CRYGS (β-crystallin S) for further verification. The results of the mass spectrometry detection for the four proteins mentioned above are shown in Table 3.

**Table 3:** Mass spectrometry detection results for CRYGS, GC, HP, and TTR

Gene Name	Protein	Average Content (ND)	Average Content (D)	Fold change	*p* value
CRYGS	Beta crystallin S	0.2581	0.0748	-3.4511	2.75E-05
GC	Vitamin D binding protein	0.2751	0.0582	-4.7251	3.98E-05
HP	Haptoglobin	0.2199	0.1125	-1.9547	7.16E-05
TTR	Transthyretin	0.1446	0.1889	1.3061	4.48E-03

ND indicated the non-diabetic control group and D indicated the diabetic experimental group

**Fig. 2:**
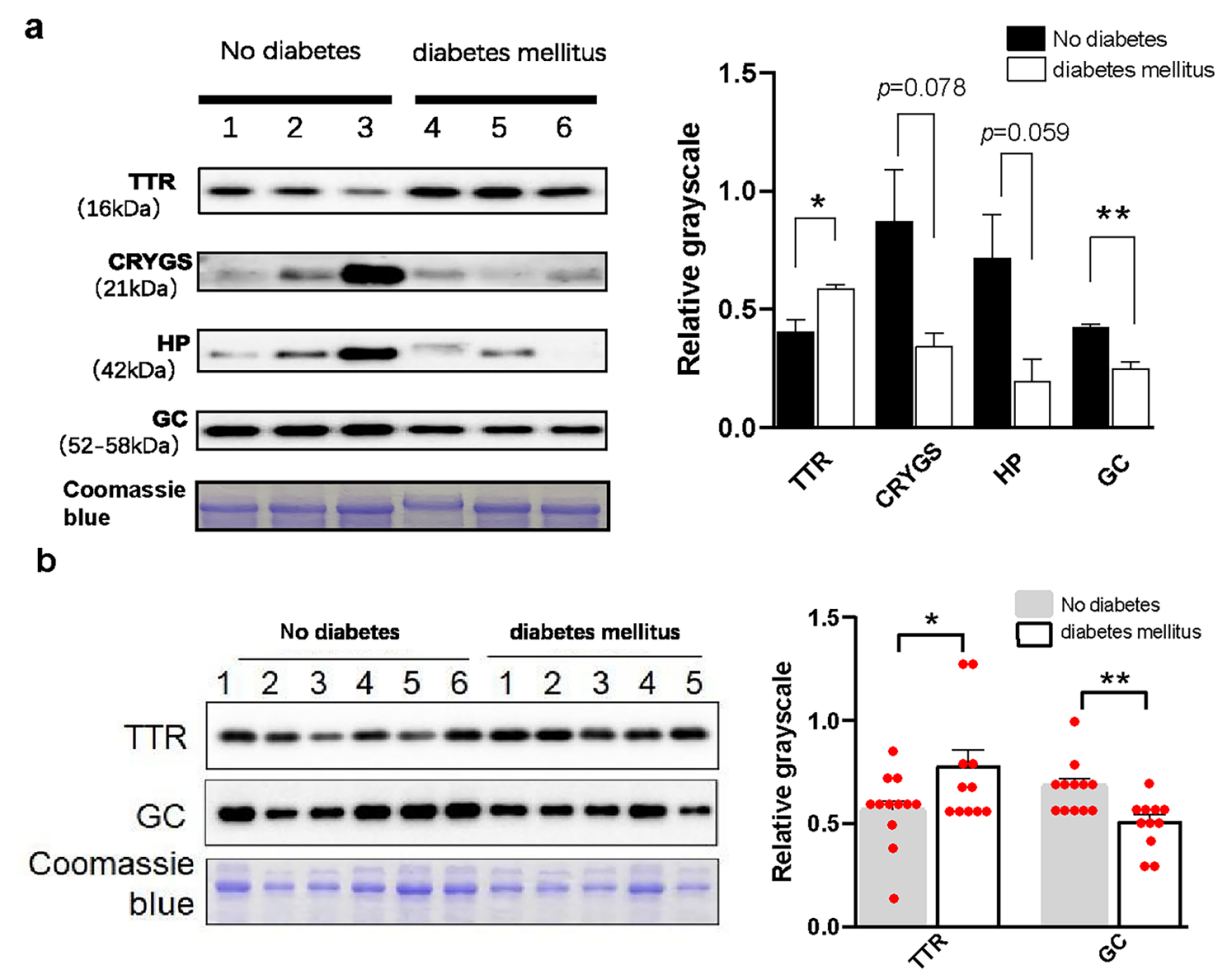
**Expression level validation of TTR, CRYGS, HP, and GC in aqueous humour protein samples**. (**a**) Each group has three independent repeats. (**b**) An expanded sample pool containing 11 diabetes patients and 12 non-diabetes patients was used. Coomassie blue was used as an internal control. Protein bands were subjected to densitometry analysis and normalized to those of Coomassie blue. *p < 0.05, **p < 0.01

The Western blot results (Fig. 2a) showed that the expression of TTR was significantly increased in the diabetes group as compared to the control while GC was significantly downregulated. There was no statistical difference in the relative expression of CRYGS and HP between the two groups (*p*-values ​​were 0.078 and 0.059, respectively). The results from the Western blot were consistent with the mass spectrometry results, indicating the reliability of the mass spectrometry results.

To further validate the expression pattern for differential proteins, we selected TTR and GC and expanded our aqueous humour sample pool to 11 patients (11 eyes) in the experimental group and 12 patients (12 eyes) in the control group. Western blot results showed that, in the experimental group, the expression of TTR was significantly upregulated while the expression of GC was significantly downregulated as compared to the control group (Fig. 2b). These results suggest that changes in TTR and GC in aqueous humour of diabetic patients have good reproducibility.

## Discussion

With the increasing prevalence of diabetes, the incidence of diabetic cataracts has also increased rapidly, and cataracts in diabetic patients are being one of the main causes of blindness and visual impairment [8, 9, 16]. People with diabetes are reported to be 2–5 times more likely to develop cataracts than people without diabetes [17].

The metabolism of the lens of the eye depends mainly on the aqueous humour, which is largely composed of water, electrolytes, organic solutes, cytokines, and proteins [18–20]. As compared to plasma, the level of protein concentration in the aqueous humour is relatively low, ranging from 150 µg/ml to 500 µg/ml [21]. However, aqueous humour proteins play an important role in maintaining the functional homeostasis of various organs and tissues in the anterior segment of the eye, including refraction, shape, and intraocular pressure [22]. The composition of the aqueous humour is influenced by adjacent tissues, and the internal environment of the eye. In patients with certain systemic diseases, including diabetes, the composition of aqueous humour about the proteins and other components change. As such, the aqueous humour has previously been used in the proteomic study of age-related macular degeneration, glaucoma, and other eye diseases [23–25].

Tandem mass spectrometry (TMT) labelling technology, when combined with high-performance liquid chromatography-mass spectrometry (HPLC-MS/MS) for high-throughput protein identification, has many advantages, including good reproducibility, high sensitivity, and data richness, making it suitable for low-volume samples like those in this study.

We separated cataract patients into two groups based on whether or not they have diabetes. It should be noted that patients with both diabetes and cataracts who participated in this study did not have fundus complications, such as retinopathy. The volumes of the aqueous humour sample we obtained from each individual patient was in the range of 0.1 ml to 0.2 ml, and the protein content was in the range of 0.2 mg to 0.6 mg, which was only 1/300-1/400 of those in plasma. The low protein content of each individual sample made it difficult to perform the proteomics study. We therefore combined samples from the same group to increase both the volume and protein content.

Although interindividual differences were present, pooling the aqueous humour samples from patients with the same groups had the advantage of reducing variability. Our mechanistic study of diabetes-related diseases focuses on the generalization of the disease in the whole population, not individual patients. In this sense, the combination of samples meets the purpose of our study.

We identified 387 proteins in human aqueous humour. Then, given the thresholds of fold change > 1.2 and *p* < 0.05, we narrowed our protein pool to 137 differential proteins, which include functional proteins, complement system proteins, signal transduction proteins, catalytic proteins, enzymes, structural proteins, and transporter proteins. Among those 137 proteins, 74 were downregulated and 63 were upregulated. We investigated their functional roles using GO enrichment analysis and KEGG pathway analysis. Through protein interaction network analysis, the 20 proteins with the greatest scores were found to be associated with inflammatory responses, metabolic diseases, organ and tissue damage. This is consistent with the occurrence and development of diabetes complications due to inflammatory reactions and oxidative stress, which suggests that the proteomic analysis of aqueous humour can provide a basis for the pathogenesis of diabetes-related cataracts. A deeper examination of the extraction using Ingenuity Pathway Analysis software indicated that the 20 selected proteins were related to retinal degeneration and diabetic complications.

The results of the Western blot validation of the four molecules, namely GC, TTR, HP, and CRYGS, obtained by the protein interaction network analysis were consistent with those of quantitative proteomics; of these, the expressions of GC and TTR showed significant differences between the two groups. Deeper validation results using an expanded sample size remained consistent with all previous data, suggesting a good level of reproducibility of this expression.

The homotetrameric carrier protein transthyretin (TTR), previously known as preambumin, is one of the three prealbumin proteins. TTR is present in all ocular structures, except the lens and tear, and can be produced by the retinal pigment epithelium [26–29]. Chen et al. (2011) found that TTR concentration is related to the pathogenesis of proliferative vitreoretinopathy [30].

Under hypoxia, TTR can induce apoptosis of the retinal microvascular endothelial cells. It can also affect the proliferation and migration of retinal microvascular endothelial cells in diabetic patients [31], suggesting that TTR may be involved in diabetic ocular complications through either self-mutation or regulating the function of the fundus microvascular endothelial cells.

The correlation between aqueous humour TTR levels and diabetes-related cataracts has not been reported. To our knowledge, this study was the first to show that TTR may be one of the key regulatory molecules for diabetic cataracts.

Vitamin D binding protein (DBP), also known as a group-specific component (GC), is a multifunctional protein that exists in plasma, ascites, cerebrospinal fluid, and on the surface of many cell types [32]. Rahman et al. (2017) suggested that the decrease in serum GC concentration could cause vitamin D insufficiency and promote the occurrence of type 2 diabetes [33]. Animal studies have shown that vitamin D supplementation could either prevent or alleviate the onset of type 2 diabetes [33]. To date, few studies have identified the presence of GC in aqueous humour.

Our study found that the decreased level of GC in aqueous humour may be involved in the pathogenesis of diabetic cataracts, suggesting that vitamin D supplementation may delay the development of diabetic ocular complications.

In conclusion, our proteomic analysis of the aqueous humour built a foundation for the mechanistic study of diabetic cataracts. Our study also revealed that TTR and GC may also serve as potential early diagnostic markers of diabetes-related cataracts and other eye complications, providing a direction for further research on these diseases and a theoretical basis for diagnosis, prevention, and treatment targets.

### Electronic supplementary material

Below is the link to the electronic supplementary material.

Supplementary Material 1

## Data Availability

The HPLC-MS/MS datasets analyzed during the current study are available from the corresponding author on reasonable request.

## Abbreviations

TMT: Tandem mass spectrometry tagging
HPLC-MS/MS: High-performance Liquid Chromatography-tandem Mass Spectrometry
FDR: False discovery rate
HP: Haptoglobin
GC: Vitamin D binding protein
TTR: Transthyretin
CRYGS: β-crystallin S
DBP: Vitamin D binding protein
