# Supplementary material for: Proteomic study of aqueous humour in diabetic patients with cataracts by TMT combined with HPLC-MS/MS
Source: BMC Ophthalmol. 2023 Oct 26;23:435. doi: 10.1186/s12886-023-03162-2 (PMC10604804; doi:10.1186/s12886-023-03162-2)
Supplement: Supplementary file 1 — Supplementary Material 1 [file 12886_2023_3162_MOESM1_ESM.docx]

**
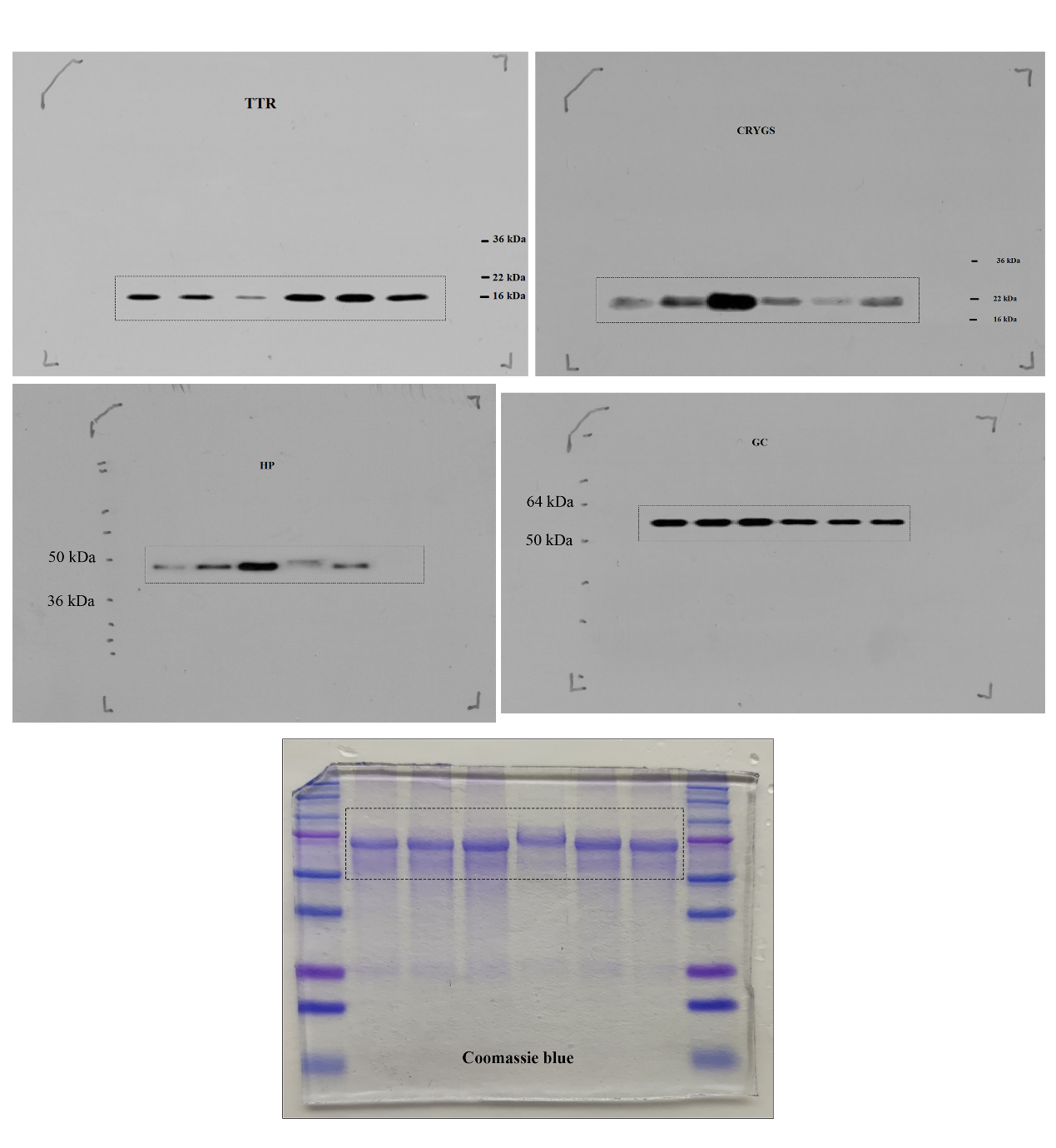
Supplementary Fig.1**. The original images for figure 2a. The exposed proteins for each gel in indicated correspondently. The dotted rectangular regions represent the cropped regions in figure 2a.

**
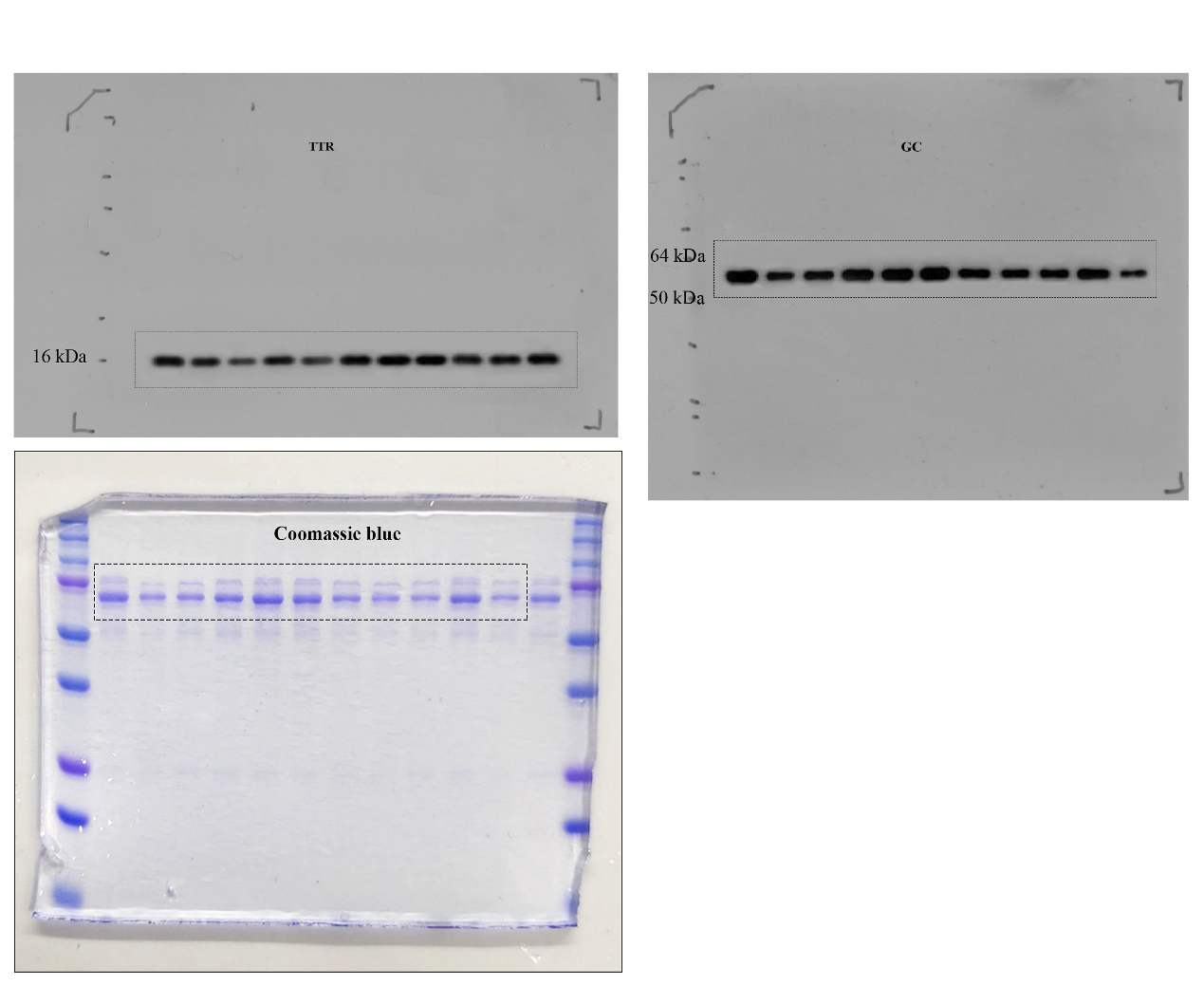
Supplementary Fig.2**. The original images for figure 2b. The exposed proteins for each gel in indicated correspondently. The dotted rectangular regions represent the cropped regions in figure 2b.
